# The enrichment of the gut microbiota Lachnoclostridium is associated with the presence of intratumoral tertiary lymphoid structures in hepatocellular carcinoma

**DOI:** 10.3389/fimmu.2023.1289753

**Published:** 2023-12-05

**Authors:** Rui Zhao, Jiacheng Li, Bo Chen, Jungang Zhao, Leyin Hu, Kate Huang, Qiwen Chen, Jiangqiao Yao, Ganglian Lin, Lishimeng Bao, Mengmeng Lu, Yi Wang, Gang Chen, Fang Wu

**Affiliations:** ^1^ Department of Gastroenterology, The First Affiliated Hospital of Wenzhou Medical University, Wenzhou, China; ^2^ Department of Hepatobiliary Surgery, The First Affiliated Hospital of Wenzhou Medical University, Wenzhou, China; ^3^ Department of Pathology, The First Affiliated Hospital of Wenzhou Medical University, Wenzhou, China; ^4^ The Second Clinical College, Wenzhou Medical University, Wenzhou, China; ^5^ Department of Epidemiology and Biostatistics, School of Public Health and Management, Wenzhou Medical University, Wenzhou, China; ^6^ Zhejiang-Germany Interdisciplinary Joint Laboratory of Hepatobiliary-Pancreatic Tumor and Bioengineering, Wenzhou, China; ^7^ Key Laboratory of Diagnosis and Treatment of Severe Hepato-Pancreatic Diseases of Zhejiang Province, The First Affiliated Hospital of Wenzhou Medical University, Wenzhou, Zhejiang, China

**Keywords:** hepatocellular carcinoma (HCC), tumor immune microenvironment (TIME), tertiary lymphoid structure (TLS), gut microbiota, Lachnoclostridium

## Abstract

**Backgrounds and aims:**

Immunotherapies have formed an entirely new treatment paradigm for hepatocellular carcinoma (HCC). Tertiary lymphoid structure (TLS) has been associated with good response to immunotherapy in most solid tumors. Nonetheless, the role of TLS in human HCC remains controversial, and recent studies suggest that their functional heterogeneity may relate to different locations within the tumor. Exploring factors that influence the formation of TLS in HCC may provide more useful insights. However, factors affecting the presence of TLSs are still unclear. The human gut microbiota can regulate the host immune system and is associated with the efficacy of immunotherapy but, in HCC, whether the gut microbiota is related to the presence of TLS still lacks sufficient evidence.

**Methods:**

We performed pathological examinations of tumor and para-tumor tissue sections. Based on the location of TLS in tissues, all patients were divided into intratumoral TLS (It-TLS) group and desertic TLS (De-TLS) group. According to the grouping results, we statistically analyzed the clinical, biological, and pathological features; preoperative gut microbiota data; and postoperative pathological features of patients.

**Results:**

In a retrospective study cohort of 60 cases from a single center, differential microbiota analysis showed that compared with the De-TLS group, the abundance of Lachnoclostridium, Hungatella, Blautia, Fusobacterium, and Clostridium was increased in the It-TLS group. Among them, the enrichment of Lachnoclostridium was the most significant and was unrelated to the clinical, biological, and pathological features of the patients. It can be seen that the difference in abundance levels of microbiota is related to the presence of TLS.

**Conclusion:**

Our findings prove the enrichment of Lachnoclostridium-dominated gut microbiota is associated with the presence of It-TLS in HCC patients.

## Highlights

• **What is already known on this topic**

Previously, the presence of intratumoral tertiary lymphoid structures (TLS) has been demonstrated to correlate with favorable immunotherapy response and long-term prognosis in patients with hepatocellular carcinoma (HCC); however, factors influencing their presence remain elusive.

• **What this study adds**

Our study has, for the first time, demonstrated the association between gut microbiota and TLS presence. We found that the enrichment of Lachnoclostridium-dominated gut microbiota is associated with the presence of intratumoral TLS in HCC a patients.

• **How this study might affect research, practice, or policy**

This provides new insights into the research on how gut microbiota affects tumor immunotherapy, specifically by modulating the formation of intratumoral TLS.

## Introduction

Primary liver cancer is the sixth most commonly diagnosed cancer type and the third leading cause of cancer death worldwide in 2020. Hepatocellular carcinoma (HCC) is the main pathological type of primary liver cancer, accounting for 75%–85% of all liver cancer cases (1). Over the past decade, the systemic therapies of tyrosine kinase inhibitors sorafenib and lenvatinib have been the first-line treatment for advanced HCC. Currently, immune checkpoint inhibitors (ICIs) and other immunotherapies are emerging as a major treatment approach in HCC due to significantly improved overall survival and lower recurrence rates in HCC patients (2–5). Despite these major advances, the full therapeutic potential of ICIs has not yet been fully realized because not all patients benefit from immunotherapy, and some HCC patients may even experience hyperprogressive disease after treatment (6, 7). Finding predictive biomarkers for good efficacy of immunotherapy can better guide the choice of clinical treatment plans, help improve patient prognosis, and solve problems pending in clinical treatment.

Tertiary lymphoid structures (TLS) are organized clusters of immune cells comprised by T and B cells and sometimes other immune cell type that develop in non-lymphoid tissues after birth. In TLS, germinal center-like aggregates of CD20+ B cells are surrounded by CD3+ T cells, resembling structures found in secondary lymphoid organs (8). TLS has emerged as a promising biomarker, as its presence in most solid tumors is closely linked with better outcomes and may predict response to ICIs (9, 10). However, the role of TLS in HCC is still debated. Recent research indicates significant functional differences depending on location within the tumor. Finkin et al. found TLS in para-tumor liver tissue increased the risk of HCC recurrence long after treatment (11). In contrast, Wolf Herman Fridman et al. found TLS within HCC tumors linked to lower odds of the cancer recurrence after surgery. They suggested that TLS inside tumors may help antitumor immunity by boosting local antigen presentation and immune cell maturation (12). Most importantly, the drivers of TLS formation in HCC and other cancers are still not fully understood.

The gut microbiota, as the largest symbiotic microbial community in humans, plays a critical role in directing the normal development of the immune system and regulating immune functions (13), including contributing to germinal center formation, regulating germinal center reactions (14), and promoting T- and B-cell activation (15, 16). Given the intertwined nature of the microbiota and the immune system, the microbiota is likely to influence the host’s response to immunotherapy. Recent clinical studies have shown that changes in the gut microbiota profiles of patients responding to immunotherapy can predict the efficacy of immunotherapy, including in HCC (17). Specific gut microbes have been identified and shown to even affect the efficacy of ICI in tumor patients, including those with gastrointestinal tumors and distal intestinal tumors (18–20). Mechanistic explorations have shown that the gut microbiota increases the number of infiltrating lymphocytes in tumor tissues. In recent years, with a deepening understanding of the function of tumor infiltrating lymphocytes (TILs), recent studies have highlighted that TILs exert their effects by forming specific spatial structures such as TLS (21). However, little is known so far about whether the gut microbiota affects the formation of specific spatial structures by TILs. Previously, research clinical features by Timothy W. Hand et al. confirmed that specific gut microbiota can support the maturation of adjacent TLS in mouse colorectal cancer (22), but further investigation is needed to determine whether similar relationships exist in human patients with tumors outside the gut.

**Figure 1:**
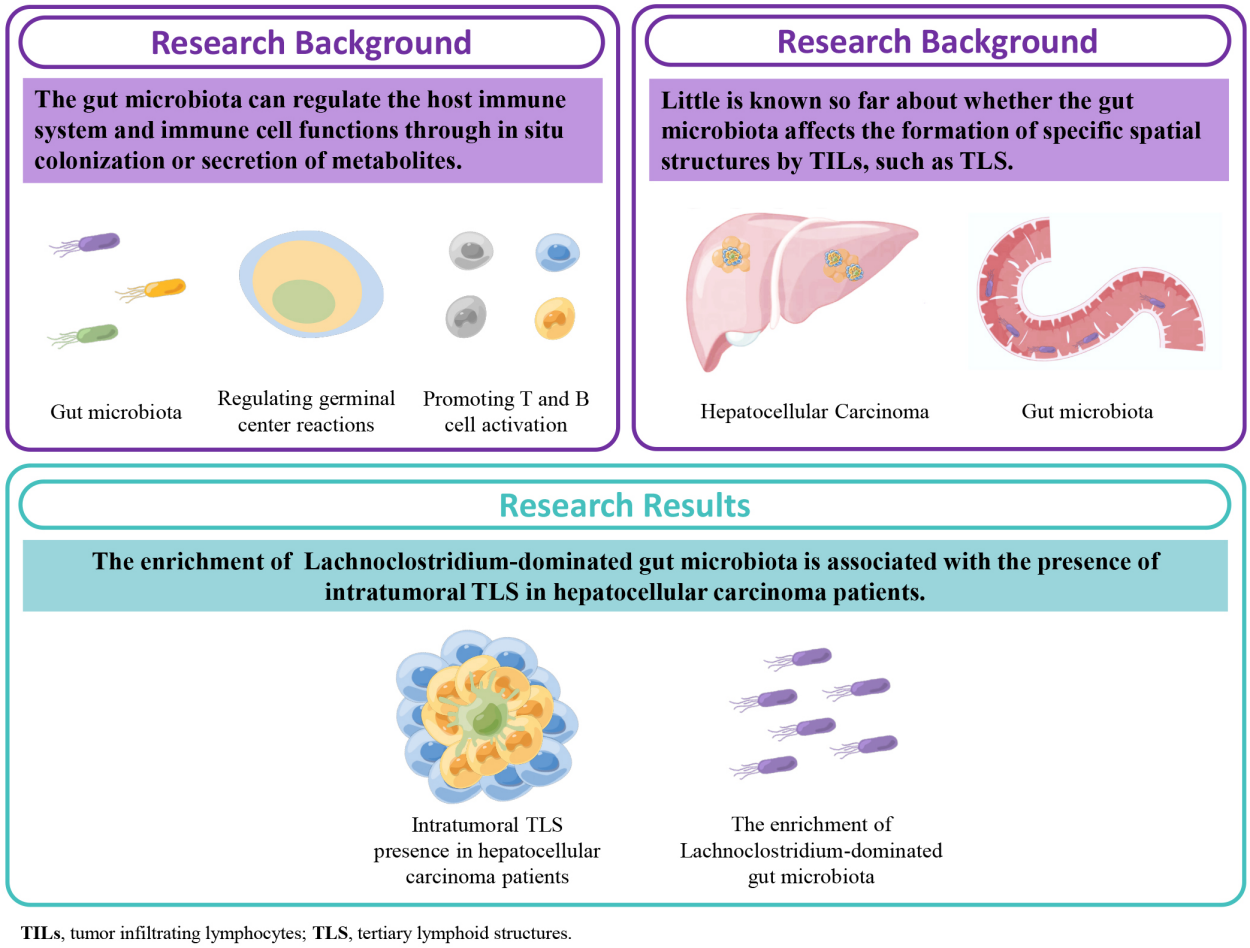
Graphical abstract, schematic diagram of this study. Our results for the first time demonstrate that the enrichment of the gut microbiota Lachnoclostridium taxa is associated with the presence of intratumoral tertiary lymphoid structures (TLS) in hepatocellular carcinoma.

In this study, we found that the enrichment of Lachnoclostridium-dominated gut microbiota is associated with the presence of intratumoral TLS (It-TLS) in HCC patients in a single-center retrospective cohort (**Figure 1**).

## Materials and methods

### Study population and specimen collection

We conducted a retrospective analysis of 60 patients who underwent curative hepatectomy for HCC at the First Affiliated Hospital of Wenzhou Medical University between 1 January 2019 and 30 June 2022. All cases were pathologically and clinically diagnosed as HCC. Within 30 min of hepatectomy, HCC tumor tissues and adjacent non-tumor tissues (defined as >3 cm from the tumor margin) were collected. The tissues were evenly cut and immediately preserved in RNA later solution. All tissue samples were stored at −80°C within 24h. The tissue specimens were obtained from the surgically resected tissues and did not cause any additional interventions or risks to the patients. Patients had not been prescribed lactulose, proton pump inhibitors, non-steroidal anti-inflammatory drugs, antibiotics, probiotics, or prebiotics within 4 weeks prior to surgery. Fecal samples were obtained from all patients before surgery for 16S rRNA sequencing analysis. This study was approved by the Ethics Committee of the First Affiliated Hospital of Wenzhou Medical University, and the study protocol conforms to the ethical guidelines of the 1975 Declaration of Helsinki.

### Clinical and biological features

We retrospectively obtained the following clinical and biological features of patients in the study cohort: age, sex, alcohol consumption (active or inactive at the time of surgery), hepatitis B virus (HBV) infection, hepatitis C virus (HCV) infection (eradicated or non-eradicated at the time of surgery), nonalcoholic fatty liver disease (NAFLD), other etiologies, Barcelona clinic liver cancer (BCLC) stage, and preoperative serum alpha-fetoprotein (AFP) levels. These clinical features were analyzed to determine their effects on the formation of TLS.

### H&E staining and multiplex immunohistochemistry

All tissues were prepared into 4-µm formalin-fixed paraffin-embedded (FFPE) sections. After dewaxing the xylene clear, the sections were deparaffinized via a series of decreasing concentrations of ethanol. The sections were then washed in deionized water and phosphate buffered saline (PBS).

For hematoxylin and eosin (H&E) staining, hematoxylin stained the nuclei and eosin stained the cytoplasm. For multiplex immunohistochemistry, antigenic epitopes were unmasked in a decloaking chamber using citrate buffer (10 mM sodium citrate and 0.05% Tween 20, pH 6). Rinsed in PBS, endogenous peroxidase activity was blocked by incubation in a 3% methanol solution of H2O2, blocked at 37°C with 5% bovine serum albumin for at least 30 min, then incubated with primary antibodies in a humidified chamber at 4°C overnight. The next day sections were incubated with anti-rabbit/mouse mixed IgG monoclonal antibodies at 37°C for 1h. Thereafter, chromogenic development was performed according to the kit manual. (Zsbio, Cat No. DS-0004).

### Multiplex immunofluorescence

All FFPE blocks prepared from patient tumor tissue and corresponding para-tumor tissue were sectioned at a thickness of 4 μm on slides. Antigen retrieval was performed on all slides as described in “IHC staining.” On the first day of the experiment, the sections were incubated with the first antibody (mouse antibody to human CD23) as described in “IHC,” and stained with fluorescein isothiocyanate/cyanine-3 (FITC/CY3) the next day. Then, the antigen epitopes were revealed again under dark conditions using citrate buffer. The sections were then exposed to primary antibodies (including rabbit antibody to human CD3 and mouse antibody to human CD20) for 16h–20h at 4°C and to secondary antibodies conjugated to Alexa 594 and Alexa 488 for 1h at room temperature. Slides were counterstained with 4’,6-diamidino-2-phenylindole to visualize cell nuclei and imaged on a Leica Stellaris 5 upright fluorescent microscope using a Leica Hyde S camera and the LAS X imaging suite.

### Pathological examination

Tumor tissue sections were strictly distinguished from para-tumor tissue sections. All sections stained with H&E were observed under microscope. The following information was recorded: tumor size, satellite nodules, invasion of large blood vessels or microvessels, multinodularity, tumor differentiation according to the World Health Organization, and non-tumorous fibrotic septa based on the METAVIR staging system.

Meanwhile, pathologists identified and categorized TLS on slides. If at least one TLS was present in the field of view of the tumor tissue, the patient was considered to have TLS within the tumor tissue, which was finally confirmed by dual immunohistochemistry and immunofluorescence. All pathological sections were evaluated separately by two pathologists specializing in liver disease (Jiacheng, Li; Leyin, Hu). Different opinions were discussed and, in case of disagreement, the final decision was made by a third senior pathologist (Kate, Huang).

### DNA extractions and PCR amplification

DNA from different samples was extracted using the cetyltrimethylammonium bromide according to manufacturer’s instructions. The full-length 16S rRNA gene was amplified using primers 27F: 5′- AGRGTTTGATYNTGGCTCAG -3′ and 1492R: 5′- TASGGHTACCTTGTTASGACTT-3′, which were tagged with specific barcode per sample. PCR amplification was performed in a total volume of 20 μL reaction mixture containing 4 μL of 5 × FastPfu Buffer, 2 μL of 2.5 mM dNTPs, 0.8 μL of each primer (5 μM), 0.4 μL of FastPfu Polymerase, and 10 ng of template DNA, and PCR-grade water to adjust the volume. The PCR conditions to amplify the FL prokaryotic 16S rRNA gene consisted of an initial denaturation at 95°C for 2 min; 25 cycles of denaturation at 95°C for 30 s, annealing at 55°C for 30 s, and extension at 72°C for 1 min; and then final extension at 72°C for 5 min.

### Library construction and sequencing

The PCR products were confirmed with 2% agarose gel electrophoresis, and purified using the AxyPrep DNA Gel Extraction Kit (Axygen Biosciences, Union City, CA, USA) according to the manufacturer’s instructions. After quantified by QuantiFluorTM-ST (Promega, Madison, WI, USA), the amplicon pools were prepared for libraries construction. SMRTbell libraries were prepared using the Pacific Biosciences SMRTbellTM Template Prep kit 1.0 (PacBio, Menlo Park, CA, USA) and sequenced on PacBio RS II (LC-Bio Technology Co., Ltd., Hangzhou, China).

### Data analysis

All clinical and biological features were translated into categorical variables, which were shown as number (percentage). Then chi-square test or Fisher exact test was performed to compare the composition differences.

Circular consensus sequence (CCS) reads were generated from raw subreads by SMRT Link (v6.0) with the following parameters: minPasses = 5; minPredictedAccuracy = 0.9. Then lima (v1.7.1) was used to distinguished CCS reads from different samples, and cutadapt (v1.9) was applied to identify primers. The CCS reads, which are between 1200 bp and 1650 bp, were remained after the length filtration. After dereplication and filtering chimeric sequences using DADA2, we obtained feature table and feature sequence. Alpha diversity and beta diversity were calculated by normalized to the same sequences randomly. Alpha diversity were applied in analyzing complexity of species diversity for a sample through five indices, including Chao1, observed species, goods coverage, Shannon, Simpson, and all these indices were calculated with QIIME2. Beta diversity was calculated by QIIME2. The ASVs were annotated by aligned feature sequences with SILVA database (release 138). Other diagrams were implemented using the R packages.

## Results

### Cohort characteristics

In our cohort of 60 patients, males comprised 83.33% and patients over 60 years of age comprised 53.33%. The primary risk factor was HBV infection in 49 patients (81.67%), followed by alcohol consumption in 21 patients (35.00%). No patient had HCV infection. 20 patients (33.33%) had multiple risk factors. According to the BCLC staging system (2022 version), two patients (3.33%) had very early stage disease (Stage 0), 49 (81.67%) had early stage disease (Stage A), nine (15.00%) had intermediate stage disease (Stage B), and zero (0.00%) had advanced stage disease (Stage C). Elevated serum AFP levels were detected in 15 patients (25.00%) (**Table 1**).

**Table 1:** Clinical, biological, and pathological features of the HCC patients according to the presence of intratumoral TLS.

Variables	All patients (*n* =35)	It-TLS (*n* = 8)	De-TLS (*n* = 27)	*P*-value
Age, > 60 years	19 (54.29%)	5 (62.50%)	14 (51.85%)	0.700
Gender, male	28 (80.00%)	7 (87.50%)	21 (77.78%)	1.000
BCLC stage, B–C	4 (11.43%)	1 (12.50%)	3 (11.11%)	1.000
AFP, > 300 ng/ml	9 (25.71%)	1 (12.50%)	8 (29.63%)	0.684
Alcohol	11 (31.43%)	3 (37.50%)	8 (29.63%)	0.685
HCV	0 (0.00%)	0 (0.00%)	0 (0.00%)	/
HBV	28 (80.00%)	4 (50.00%)	24 (88.89%)	0.033
NAFLD	0 (0.00%)	0 (0.00%)	0 (0.00%)	/
Other etiology	6 (17.14%)	3 (37.50%)	3 (11.11%)	0.117
PS score, 1–2	22 (62.86%)	3 (37.50%)	19 (70.37%)	0.116
Tumor size, > 5cm	15 (42.86%)	2 (25.00%)	13 (48.15%)	0.419
Satellite nodules	8 (22.86%)	1 (12.50%)	7 (25.93%)	0.684
Microvascular invasion	16 (45.71%)	4 (50.00%)	12 (44.44%)	1.000
Poor differentiation	4 (11.43%)	0 (0.00%)	4 (14.81%)	0.553
Cirrhosis	22 (62.86%)	3 (37.50%)	19 (70.37%)	0.116

Statistical analysis was performed using chi-square tests. AFP, alpha-fetoprotein; BCLC, Barcelona Clinic Liver Cancer; HBV, hepatitis B virus; HCV, hepatitis C virus; NAFALD, Nonalcoholic fatty liver disease; TLS, tertiary lymphoid structure.

### Pathological findings and cohort grouping

**Figure 2:**
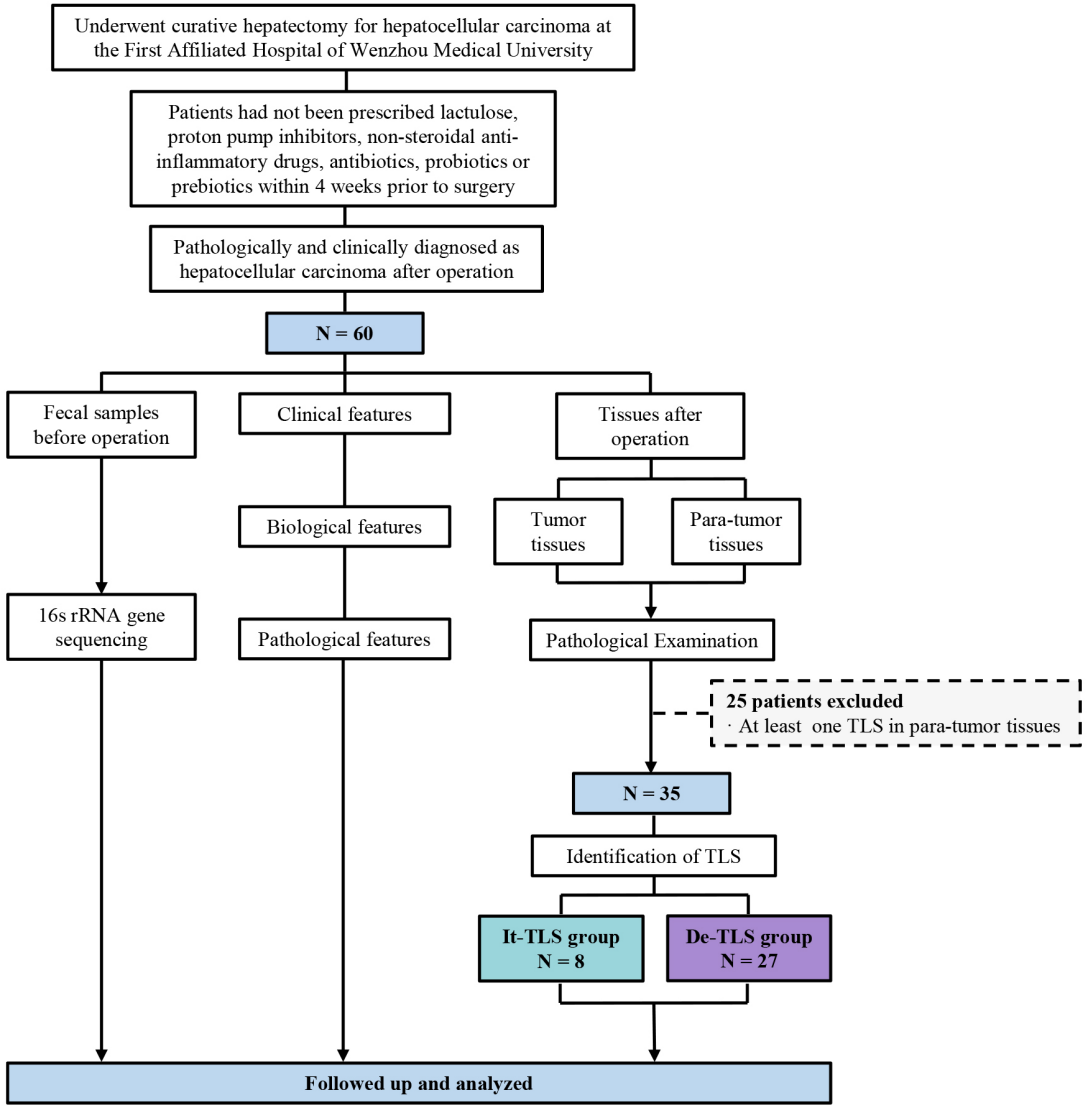
The flowchart of this study. TLS, tertiary lymphoid structure; It-TLS group, Intratumoral TLS group; De-TLS group, Desertic TLS group.

In a retrospective cohort of 60 patients, we found TLS in 33 cases (55.00%). Given previous studies showing that TLS located in para-tumor parenchyma was associated with increased late recurrence risk in HCC and could serve as an ecological niche to maintain the survival of transformed hepatocytes (11), we further distinguished para-tumor TLS in cases with TLS. Among these, TLS was observed only within tumor tissues in eight cases (13.33%) but not in para-tumor tissues. These were identified as tumor tissue only (It-TLS group). In 27 cases (45.00%), no TLS was observed in either tumor or para-tumor tissues (desertic-TLS group, De-TLS). The grouping of this study is shown in **Figure 2**.

### Identification of tertiary lymphoid structures

**Figure 3:**
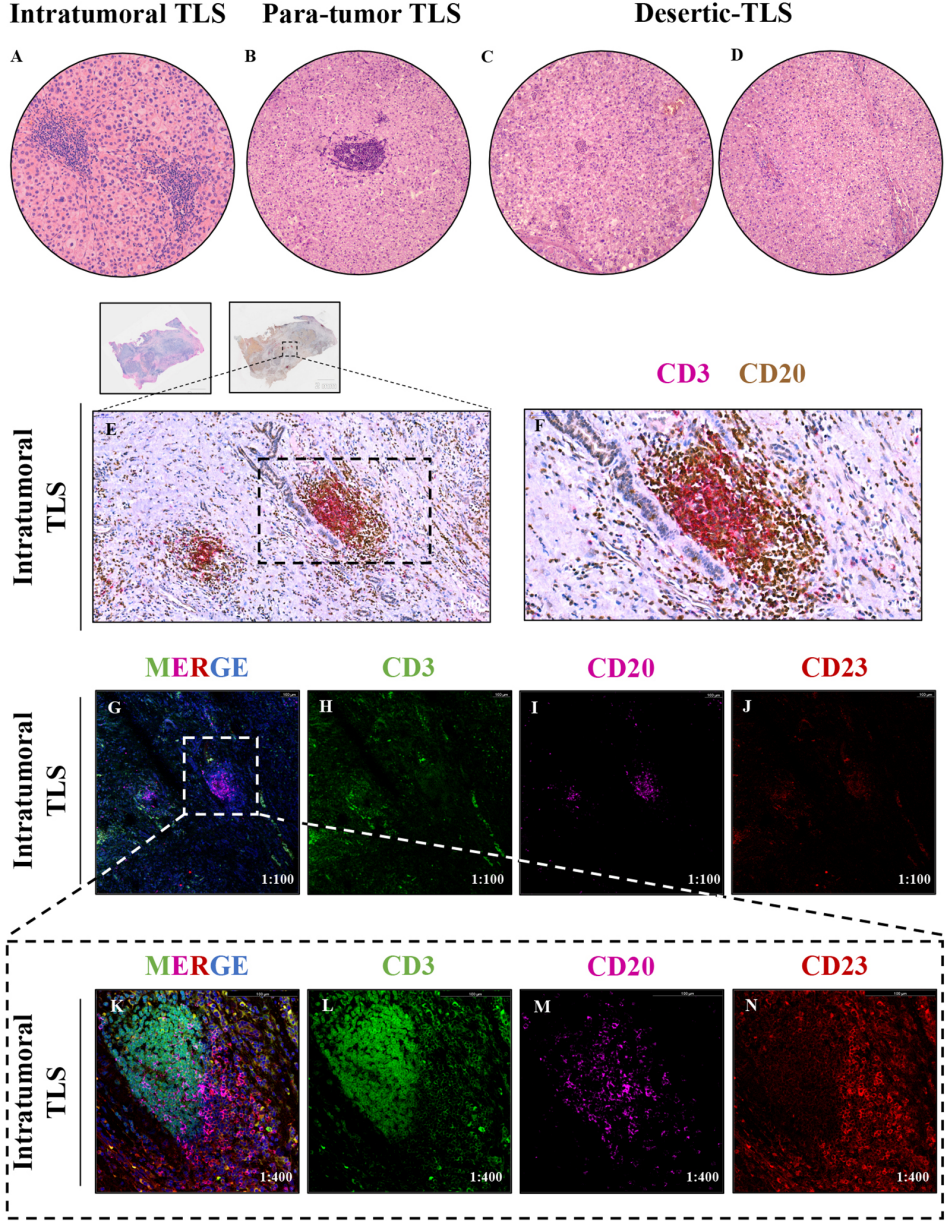
**(A–D)** The tertiary lymphoid structures (TLS) in tumor and para-tumor tissues of hepatocellular carcinoma were identified and grouped by H&E staining,1:200. **(A)** Tertiary lymphoid structure in tumor tissue, namely, intratumoral TLS group (It-TLS) group. **(B)** Tertiary lymphoid structure exists in para-tumor tissues. **(C, D)** TLS is not found in tumor tissue and para-tumor tissues, namely, desertic TLS group, De-TLS group. **(E, F)** Mature tertiary lymphoid structure in tumor tissue by multiplex immunohistochemistry, CD3+ T cells, CD20+ B cells, E 1:200, F 1:400. **(G–J)** CD3+ T cells, CD20+ B cells, and CD23+FDCs multiplex immunofluorescence, 1:100; **(K–N)** Representative regions CD3+ T cells, CD20+ B cells and CD23+FDCs multiplex immunofluorescence, 1:400.

Two hepatopathologist examined tumor sections and para-tumor sections under microscopy. According to the recent expert consensus (23), dense lymphoid aggregates within the liver parenchyma containing ≥50 immunocyte nuclei were preliminarily identified as TLS, It-TLS was required to be surrounded by and/or embedded within the tumor matrix. We confirmed the presence of TLS using multiplex immunohistochemistry. CD3 was utilized to label peripheral T cells within the TLS, while CD20 was used to label B cells within the TLS. To further verify that the observed structures were TLS, we performed immunofluorescence staining on all sections containing presumed TLS and assessed the maturity of TLS by CD23 staining (**Figure 3**).

### The presence of intratumoral TLS was unrelated to cohort characteristics

We performed statistical analyses between the It-TLS group and De-TLS. The results showed that all clinical, biological, or pathological features did not differ between HCC patients with or without It-TLS (It-TLS vs. De-TLS group, *p* > 0.05, chi-squared test) (**Table 1**). These findings indicate that underlying liver disease did not influence the presence of It-TLS.

### Alterations in microbiota were associated with the presence of intratumoral TLS

**Figure 4:**
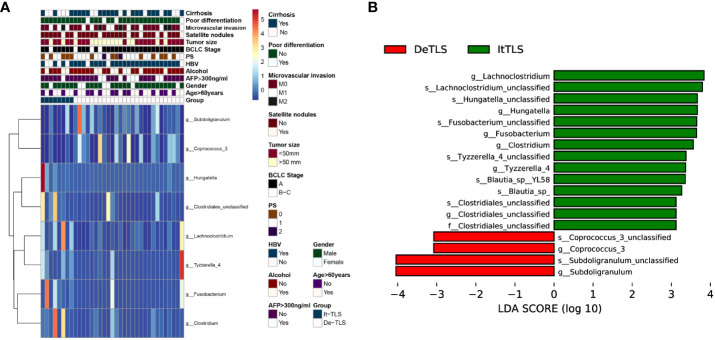
**(A)** Heatmaps of differential clinical, biological, and pathological features characteristics and flora features of HCC patients between It-TLS group and De-TLS group in our cohort, the heatmap was generated using the pheatmap R package. **(B)** Differential flora between It-TLS group and De-TLS group.

Based on the above grouping, we performed 16s rRNA sequencing. Observed species and Chao1 were used to assess species richness. Shannon and Simpson reflected species abundance and evenness. Our results showed that there were no significant differences in α diversity between the It-TLS and De-TLS groups. The analysis of β diversity within groups showed no significant differences in the It-TLS and De-TLS groups. Analysis of the differentially abundant genera suggested that compared with the De-TLS group, the abundance of Lachnoclostridium, Hungatella, Blautia, Fusobacterium, Clostridium, Tyzzerella, and Clostridiales increased in the It-TLS group. Among them, the enrichment of Lachnoclostridium was the most significant. Collectively, these results indicate that differences in the abundance of microbiota were associated with the presence of TLS (**Figure 4**). We have consolidated the results pertaining to the comparison of microbiota data between the It-TLS group and the De-TLS group. The relevant data are accessible in **Supplementary Figure S1**.

## Discussion

Immunotherapy has produced unprecedented durable therapeutic responses in HCC and other solid tumors, bringing revolutionary changes to cancer treatment (2). This clinical outcome has aroused people’s interest in exploring immune components in the tumor microenvironment, namely, the tumor immune microenvironment (TIME) (24). TIME is closely related to tumor development, recurrence, and metastasis. As research deepens, some unexplained results have also emerged, for example, earlier studies focusing on cellular components in TIME showed that patients with similar immune cell infiltration had different prognoses (25), suggesting the necessity to explore the spatial structure of TIME in tumors to further deepen understanding of the impact of TIME on tumors. With the study of the spatial distribution of immune cells within tumors, some aggregation patterns of immune cells have attracted attention due to their functional consistency in multiple tumor types and different individuals and potential clinical value, such as TLS (26). Since the back-to-back studies published in Nature successfully demonstrated that TLS affected the objective response rate of ICB in melanoma and was associated with good patient prognosis (10, 27), similar conclusions have been obtained in most tumors (28–30). In particular, in a previous study by Mark Yarchoan, cabozantinib and nivolumab converted locally advanced HCC into a resectable disease, and significant enrichment of TLS was present in the tumor tissue of responders (31). Recently, Fridman et al’s study showed that the presence of TLS in advanced soft tissue sarcoma could be used as a predictive biomarker to improve patients’ drug selection for pembrolizumab treatment (32). These findings demonstrate the application prospect of TLS in helping patients choose clinical treatment regimens. To encapsulate, this seems to form a guideline for the “clinical benefit” of TLS in the tumor context.

Interestingly, this guideline is currently controversial in HCC: initially, Finkin et al’s study challenged this tenet. Their study found that ectopic TLS in HCC provided a growth environment for malignant hepatic progenitor cells (11), recently, Wenjie Song et al’s study found that CD15+ neutrophil infiltration in HCC, and increased density of TLS around the tumor were associated with worse prognosis (33). Fridman et al’s observed in early HCC that immature TLS formation was associated with overexpression of genes related to immunosuppression, immune failure, and tumor immune escape, promoting tumor immune evasion (34). However, Hong Wu et al’s previous study showed that para-tumor TLS were associated with improved patient prognosis (35). Specifically, they found decreased infiltration of FOXP3+, CD68+, and PD1+ cells in para-tumor TLS. Valerie Chew et al’s work showed that close interaction between tumor-infiltrating T cells and B cells was associated with enhanced local immune activation and contributed to better prognosis in HCC patients (36), supporting Fridman et al’s finding that It-TLS in HCC had good clinical prognostic value (12). In summary, given the contradictory results of TLS on anti-tumor immunity in HCC, studying the specific cellular composition and origin of TLS in HCC may provide more effective information. Nonetheless, the factors influencing the formation of TLS in HCC are currently unclear, which greatly limits the exploration of TLS in HCC. Therefore, there is an urgent need to clarify the factors affecting the formation of It-TLS in HCC.

The microbiota in the host gut can regulate the host immune system. The role of the gut microbiota in the progression of gastrointestinal tumors is undoubtedly crucial. In particular, a recent series of studies have shown that the gut microbiota is involved in and affects anti-tumor immunity, including regulating patients’ clinical responses to ICIs. The exact mechanisms by which the gut microbiota influences cancer immunotherapy are being gradually revealed, surprisingly not only in gastrointestinal tumors but also including pancreatic cancer and melanoma (19, 20). Previously, Giandomenica Iezzi et al’s work demonstrated that microbiota abundance and high chemokine expression were associated with TILs recruitment (37). As a specific spatial form of TILs, Helicobacter hepaticus can promote TLS maturation in mouse CRC models (22). However, whether the gut microbiota is associated with TLS in HCC still lacks sufficient evidence.

**Figure 5:**
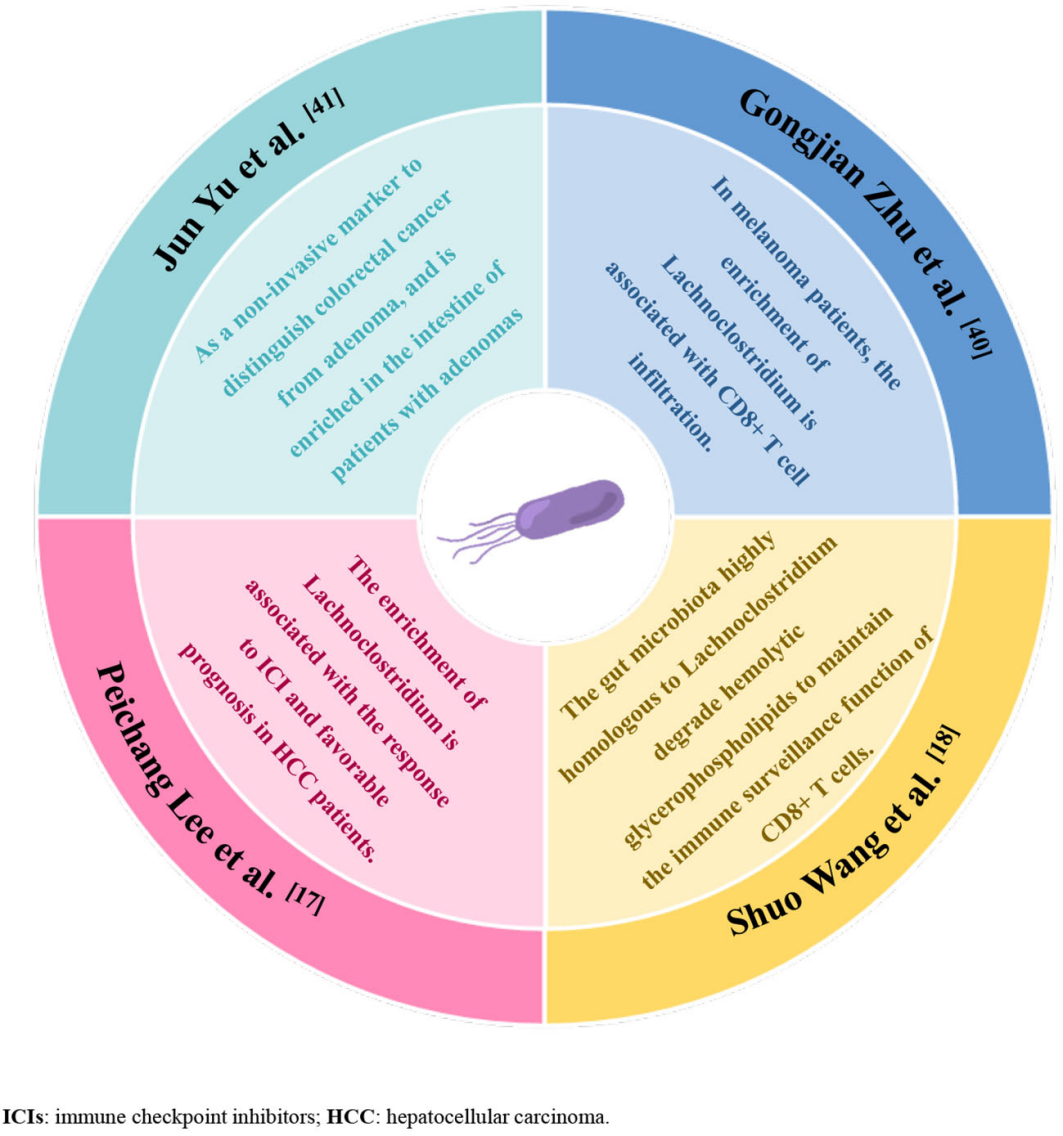
Graphical abstract, summary of the evidence from previous studies supporting that enrichment of Lachnoclostridium-dominated gut microbiota promotes anti-tumor immunity.

To our knowledge, this is the first study to explore the relationship between gut microbiota and It-TLS in extraintestinal tumors. Our study shows that the presence of TLS in tumor tissues of HCC patients is associated with enrichment of specific gut microbial phyla, specifically, increased enrichment of Lachnoclostridium, Hungatella, Fusobacterium, and Clostridium in these patients, among which Lachnoclostridium enrichment in the tumor TLS group was most pronounced. Lachnoclostridium belongs to the family Lachnospiraceae (38). Although intestinal microbiota members belonging to the Lachnospiraceae family have been shown to play important roles in regulating the host’s immune system, our understanding of the functional diversity of strains belonging to this family remains incomplete. Recently, Shuo Wang et al. found that Ruminococcus gnavus, a member of the Lachnospiraceae family, can act as an intratumoral bacterium to increase and degrade hemolytic glycerophospholipids that inhibit CD8+ T-cell activity. Maintaining CD8+ T-cell immune surveillance, thereby reducing colon tumor growth (18), and Lachnoclostridium is highly homologous to Ruminococcus gnavus (39). Previously, Peichang Lee et al. first demonstrated the important role of the gut microbiome-liver axis in the therapeutic response and survival of ICI treatment in HCC patients. In addition, in responders to immunotherapy with unresectable HCC, increased enrichment of Lachnoclostridium was found in fecal samples, which was associated with better overall survival (17), suggesting an important role of Lachnoclostridium in immunotherapy for HCC. In addition, analysis of the intratumoral microbiome in melanoma showed that Lachnoclostridium was positively correlated with the number of CD8+ T-cell infiltration in tumor tissues and affected patient survival (40). Lachnoclostridium has also been reported as a non-invasive marker to distinguish colorectal cancer from adenoma and is enriched in the intestine of patients with adenomas (41). Although our study has not yet revealed the specific mechanism by which Lachnoclostridium influences the presence of TLS at the experimental animal level, published studies may allow us to speculate that Lachnoclostridium may affect lymphocyte recruitment or activation in HCC and promote TLS formation within tumor tissues, but this requires further validation (**Figure 5**).

For other differential flora between the It-TLS group and the De-TLS group, we focused on several sub-high abundance flora, including Hungatella, Fusobacterium, and Clostridium in addition to Lachnoclostridium. In a previous study of differential flora between colorectal adenomas and colorectal cancer, Hungatella hathewayi was enriched in the colorectal cancer group (42). In addition, previous studies have reported that Hungatella increased in cancer patients who did not respond to anti–PD-1 and chemotherapy combined treatment (43). Fusobacterium has recently been defined as a “notorious” expert in cancer immunotherapy by many studies. Jingyuan Fang et al. showed that succinic acid derived from F. nucleatum inhibited the cGAS–interferon-β pathway, thereby inhibiting anti-tumor responses by limiting the transport of CD8+ T cells (44), while Shuo Wang ‘s study found that Fusobacterium nucleatum can limit the function of Lachnospiraceae and promote tumor progression (18). In HCC tumor tissues, the enrichment of Fusobacterium was accompanied by a significant increase in processes such as fatty acid and lipid synthesis, which is thought to be a key factor in the effect of intratumoral microbes on tumor progression (45). Clostridium XIVa was enriched in the intestinal flora of patients with HBV-related HCC with high tumor burden and may affect disease progression through bile acid metabolism (46). To encapsulate, several other differential flora did not seem to show the function of promoting anti-tumor immunity in the body, but rather the ability to promote tumor progression, but their role in HCC still needs more experiments to prove. Finally, although we observed a significant enrichment of Subdoligranulum in the De-TLS group, we did not find studies on its mechanism of function in tumors. Instead, it has been more reported in inflammatory diseases and is related to regulating the function of Th17 cells (47).

In this single-center retrospective study, most of the patients had HBV-related HCC, without the other two common underlying chronic liver diseases of HCC–HCV infection and NAFLD. Therefore, our data may be more applicable to HBV-related HCC. In addition, our data also showed no statistical differences in either clinical or pathological features between It-TLS and De-TLS groups, which supports microbiota difference as an independent impact factor for TLS existence. However, some data may need further exploration. We found less than half of patients with It-TLS had liver cirrhosis, while over 70% patients in De-TLS group had cirrhosis (*p* = 0.1). Previous studies have shown decreased immune cell infiltration in HCC tissues of cirrhotic mice (48). However, there are few studies evaluating It-TLS formation in human HCC patients with liver cirrhosis, which could be a potential direction for future research.

In conclusion, our results for the first time demonstrate that the enrichment of the gut microbiota Lachnoclostridium taxa is associated with the presence of It-TLS in HCC. Our study provides a new line of reasoning for the mechanism by which gut microbes influence cancer immunotherapy, that is, by affecting the formation of specific spatial structures of tumor-infiltrating lymphocytes–TLS, thereby promoting anti-tumor immunity. We show that the gut microbiota may be an interesting research focus. In the future, Related studies in experimental animals may have a positive impact on revealing the mechanism by which gut microbes regulate the formation of TLS in HCC tumors.

## Limitation of the study

Regrettably, there are still some limitations in this study. First, due to the short follow-up time, we did not analyze the prognosis of patients, although previous studies have supported it, further study of the specific mechanisms is warranted. Second, due to the limitations of sequencing technology, we were unable to finely identify some differential flora. Finally, we did not study the causal relationship between changes in the intestinal microbiome and the existence of TLS in tumors. Further study of the mechanisms is essential to elucidate the exact interactions between gut microbiota and It-TLS, as well as identify potential therapeutic targets. In future studies, we plan to further confirm our view in a mouse model of primary hepatocellular carcinoma.

## Data availability statement

The datasets presented in this article are not readily available because the privacy of the patients involved in the study needs to be protected, and the 16S rRNA sequencing data in the research is not readily available. Requests to access the datasets should be directed to Rui Zhao, wzykdx0412@126.com.

## Ethics statement

The studies involving humans were approved by The ethics committee of the First Affiliated Hospital of Wenzhou Medical University. The studies were conducted in accordance with the local legislation and institutional requirements. The participants provided their written informed consent to participate in this study.

## Author contributions

RZ: Writing – original draft, Writing – review & editing, Conceptualization, Data curation, Methodology, Project administration, Visualization. JL: Writing – original draft, Writing – review & editing, Conceptualization, Data curation, Methodology, Project administration. BC: Writing –original draft, Writing – review & editing, Conceptualization, Software, Project administration. JZ: Writing – original draft, Writing – review & editing, Conceptualization, Project administration. LH: Writing – original draft, Writing –review & editing, Visualization, Data curation. KH: Writing – review & editing, Visualization, Data curation. QC: Writing –review & editing, Data curation. JY: Writing – review & editing, Data curation. GL: Writing – review & editing, Data curation. LB: Writing – review & editing, Data curation. ML: Writing – review & editing, Data curation. YW: Writing – original draft, Writing – review & editing, Funding acquisition. GC: Writing – original draft, Writing – review & editing, Resources, Conceptualization, Supervision, Funding acquisition. FW: Writing –original draft, Writing – review & editing, Resources, Conceptualization, Supervision, Funding acquisition.

## Supplementary material

Supplementary Figure 1 The 16S rRNA data of the It-TLS group and De-TLS group were analyzed. Firstly, microbiota compositional analyses were undertaken. A stacked bar chart **(A)** delineated the top 30 species with the highest relative abundances at the genus level across different samples. A Venn diagram **(B)** depicted the species compositional similarities and overlaps between the two groups at the genus level. A Circos plot **(C)** visualized sample-species co-occurrence at the genus level, elucidating the correspondence between samples and species. Subsequently, analyses of microbiota diversity were conducted. Regarding α-diversity metrics, Observed species **(D)** and Chao1 **(E)** reflected species richness in samples irrespective of proportional abundances, whereas Shannon **(F)** and Simpson **(G)** indexes reflected both species richness and evenness. For β-diversity metrics, PCA **(H)** and PCoA **(I)** revealed variation in community compositions, and NMDS analysis **(J)** revealed differences among individuals. Finally, differential abundance analysis was performed to ascertain differentially abundant taxa. Fisher’s exact test **(K)** identified differentially abundant microbiota. Taken together, the results demonstrate distinct gut microbiota between the It-TLS and De-TLS groups, with enrichment of Lachnoclostridium in the It-TLS group. All analyses were performed on the OmicStudio (https://www.omicstudio.cn/home).
