# Supplementary figures and images for: The enrichment of the gut microbiota Lachnoclostridium is associated with the presence of intratumoral tertiary lymphoid structures in hepatocellular carcinoma
Source: Front Immunol. 2023 Dec 5;14:1289753. doi: 10.3389/fimmu.2023.1289753 (PMC10728494; doi:10.3389/fimmu.2023.1289753)

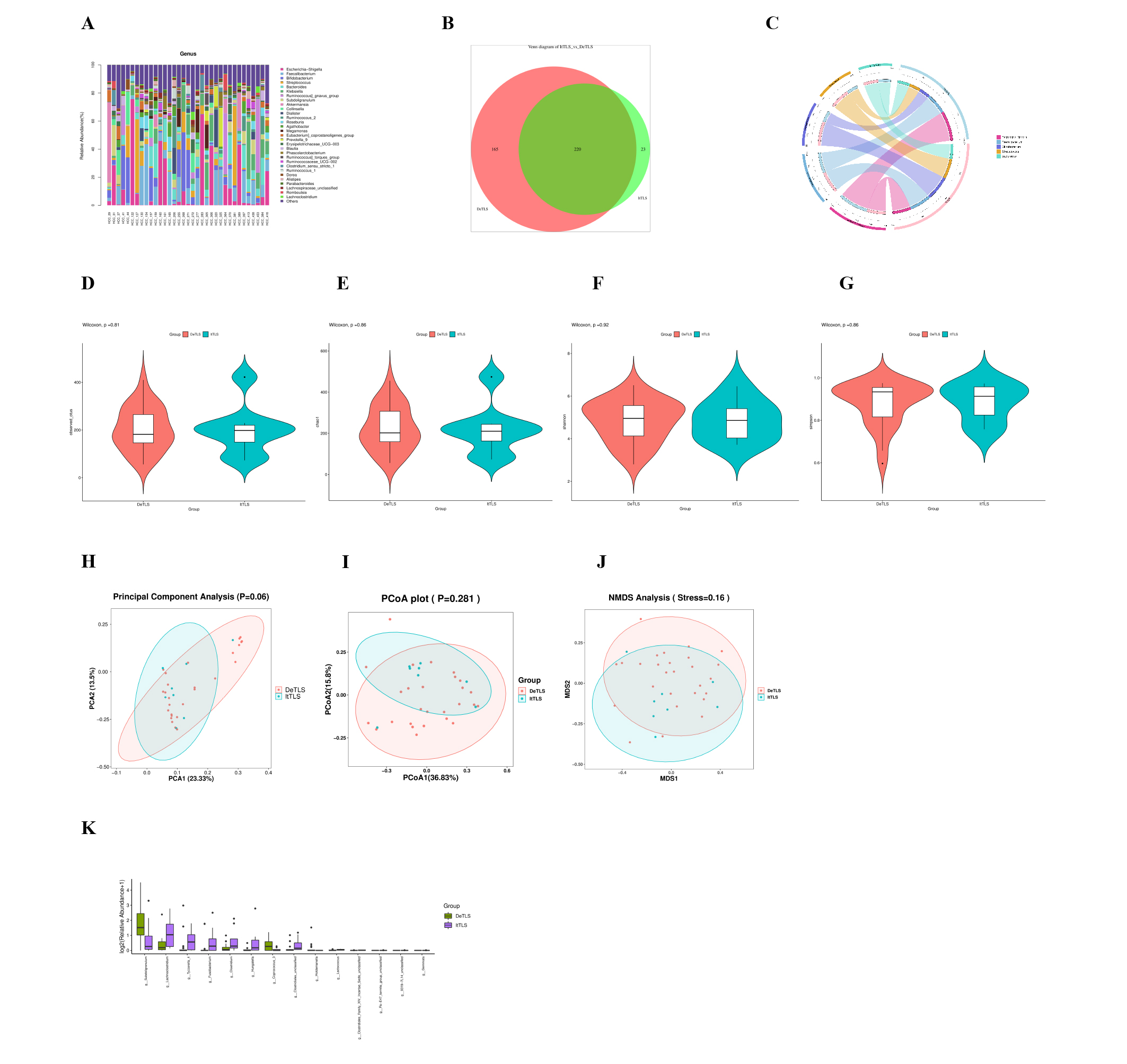

Supplement: Supplementary Figure 1 — The 16S rRNA data of the It-TLS group and De-TLS group were analyzed. Firstly, microbiota compositional analyses were undertaken. A stacked bar chart (A) delineated the top 30 species with the highest relative abundances at the genus level across different samples. A Venn diagram (B) depicted the species compositional similarities and overlaps between the two groups at the genus level. A Circos plot (C) visualized sample-species co-occurrence at the genus level, elucidating the correspondence between samples and species. Subsequently, analyses of microbiota diversity were conducted. Regarding α-diversity metrics, Observed species (D) and Chao1 (E) reflected species richness in samples irrespective of proportional abundances, whereas Shannon (F) and Simpson (G) indexes reflected both species richness and evenness. For β-diversity metrics, PCA (H) and PCoA (I) revealed variation in community compositions, and NMDS analysis (J) revealed differences among individuals. Finally, differential abundance analysis was performed to ascertain differentially abundant taxa. Fisher’s exact test (K) identified differentially abundant microbiota. Taken together, the results demonstrate distinct gut microbiota between the It-TLS and De-TLS groups, with enrichment of Lachnoclostridium in the It-TLS group. All analyses were performed on the OmicStudio (https://www.omicstudio.cn/home). [file Image_1.jpeg]
